# S100A8/A9 as a biomarker for differentiating pulmonary tuberculosis from community-acquired pneumonia in children: a multicenter cohort study in China

**DOI:** 10.3389/fped.2026.1910674

**Published:** 2026-10-01

**Authors:** Xiaoxue Li, Xu Li, Min Fang, Xiaomei Liu, Li Duan, Ming Li, Yanan Fu, Yanchun Wang, Li Jiang, Xiaotao Yang, Tingting Jiang, He Tang, Li Tan, Meifen Wang, Yang Liu, Yu Zhu, Jing Bi, Adong Shen

**Affiliations:** 1 Beijing Key Laboratory of Pediatric Respiratory Infection Diseases, Key Laboratory of Major Diseases in Children, Ministry of Education, National Clinical Research Center for Respiratory Diseases, Laboratory of Respiratory Diseases, Beijing Pediatric Research Institute, Beijing Children's Hospital, Capital Medical University, National Center for Children's Health, Beijing, China; 2 The No. 7 People's Hospital of Liangshan Prefecture, Liangshan, China; 3 Kunming Children’s Hospital, Kunming Medical University, Kunming, China; 4 Baoding Key Laboratory for Precision Diagnosis and Treatment of Infectious Diseases in Children, Hebei Key Laboratory of Infectious Diseases Pathogenesis and Precise Diagnosis and Treatment, Baoding Hospital of Beijing Children's Hospital, Capital Medical University, Baoding, China; 5 West China Second Hospital, Sichuan University, Chengdu, China

**Keywords:** biomarker, children, community-acquired pneumonia, S100A8/A9, tuberculosis

## Abstract

**Background:**

Differentiating pulmonary tuberculosis (PTB) from community-acquired pneumonia (CAP) in children remains a significant diagnostic challenge. This study aimed to evaluate the diagnostic value of serum S100A8/A9 in pediatric populations.

**Methods:**

This multicenter prospective study enrolled 1,629 children aged 0∼≤ 18 years with definitive diagnostic classifications, including a discovery cohort (*n* = 1,092) and a validation cohort (*n* = 537). Participants were stratified into three groups: PTB (Confirmed, Unconfirmed), CAP (Viral, Bacterial, *Mycoplasma pneumoniae*, Mixed), and healthy controls (HC). Serum S100A8/A9 levels were quantified by enzyme-linked immunosorbent assay, and diagnostic performance was assessed using ROC analysis.

**Results:**

In both cohorts, S100A8/A9 levels were markedly lower in PTB than in CAP but higher than in healthy controls. (both *p* < 0.001). For distinguishing PTB from CAP, S100A8/A9 achieved AUC values of 0.916 (95% CI: 0.895-0.937, sensitivity 87.1%, specificity 81.8%) in the discovery cohort and 0.932 (95% CI: 0.906–0.958, sensitivity 87.5%, specificity 86.4%) in the validation cohort. Compared with all CAP etiological subgroups, PTB children had the lowest S100A8/A9 levels, with the highest AUC of 0.954 for PTB vs. *Mycoplasma pneumoniae* CAP. Age-stratified analysis revealed excellent discriminatory performance in children aged 5-18 years (AUC = 0.955 for PTB versus overall CAP). but impaired in children under 5 years (AUC = 0.838 for PTB versus overall CAP), with lower sensitivity (57.4%) for discriminating PTB from Viral-CAP. Furthermore, a concentration-dependent distribution was observed, with higher S100A8/A9 levels associated with a decreasing proportion of PTB and an increasing proportion of CAP. In children aged 5–18 years, levels < 1,000 ng/mL were predominantly PTB (92.1%), and levels > 1,900 ng/mL were highly predictive of CAP (96.8%).

**Conclusions:**

Serum S100A8/A9 is a promising potential biomarker for distinguishing pediatric PTB from CAP, characterized by age-related distribution and concentration-dependent diagnostic pattern.

## Introduction

Tuberculosis (TB), attributable to *Mycobacterium tuberculosis* (MTB), continues to be a leading cause of morbidity and mortality among children globally. According to the World Health Organization (WHO), an estimated 1.20 million children aged 0-14 years developed TB in 2024, while only 58% of cases were reported (1). Diagnostic challenges in childhood TB largely explain this gap in case detection (2). Despite finding bacteria in clinical samples being the gold standard for TB diagnosis, its sensitivity is less than 40% in pediatric TB due to difficulties in sample collection and low bacterial load (3). Without definitive microbiological confirmation, pediatric TB diagnosis relies on clinical symptoms and radiographic evidence. However, as the most common TB disease type, pulmonary TB (PTB) shares similar clinical manifestations (e.g., fever, cough, and respiratory distress) and radiographic features to community-acquired pneumonia (CAP), the most common respiratory infection, often resulting in misdiagnosis or delayed diagnosis (4, 5). Approximately 12.6–32.1% of cases, an initial diagnosis of CAP is revised to PTB following further investigation (6, 7). Timely and accurate differentiation of PTB from CAP is critical to avoid inappropriate therapy, stop disease progression, and minimize PTB transmission risk. Thus, novel non-invasive biomarkers with high diagnostic accuracy are urgently needed for distinguishing pediatric PTB from CAP.

S100A8/A9, also referred to as calprotectin, belongs to the S100 family of proteins and plays a pivotal role in regulating inflammatory responses and host defense against infections (8). Studies have shown that S100A8/A9 promotes PTB pathogenesis by enhancing neutrophil recruitment and aggravating pulmonary inflammation (9, 10), and its serum levels correlate with disease activity (10). Interestingly, evidence indicates that S100A8/A9 is a potential biomarker for assessing severity and guiding treatment strategies in CAP (11). Nevertheless, few studies have systematically evaluated the diagnostic value of S100A8/A9 for differentiating PTB from CAP in children, and existing data are limited by small sample sizes, single-center designs, and a lack of subgroup analyses across different age groups and CAP etiologies.

To address these knowledge gaps, we conducted a prospective, multicenter study involving two independent cohorts. The objectives of this study were to assess the diagnostic performance of serum S100A8/A9 in differentiating pediatric PTB from HC and CAP and validate in an independent cohort.

## Methods

### Study design and population

This was a multicenter prospective study conducted in four hospitals across three provinces in China, including Baoding Hospital of Beijing Children's Hospital (Hebei Province), Kunming Children's Hospital (Yunnan Province), the 7th People's Hospital of Liangshan Prefecture, and West China Second Hospital of Sichuan University (Sichuan Province). Two independent cohorts were enrolled consecutively following the same study protocol: discovery cohort was recruited from September 2022 to September 2024, and validation cohort was subsequently enrolled from October 2024 to October 2025 from the same study sites.

Eligible participants were children aged 0 ∼ ≤ 18 years who presented with respiratory signs or symptoms suggestive of PTB or CAP (e.g., fever, cough, and other respiratory manifestations). Definitive classification of PTB and CAP was made in accordance with the corresponding Chinese and international diagnostic guidelines (12–14). Additionally, healthy children (HC) who underwent routine physical examination and had no evidence of infectious, inflammatory, or immunological diseases and interferon-*γ* release assays (IGRA) negative were enrolled as the HC group.

Exclusion criteria were as follows: (1) not meet the inclusion criteria; (2) prior anti-tuberculosis treatment for over 2 weeks; (3) prior antibiotic administration for over 72 h; (4) presence of immunosuppressive or immunodeficiency diseases (e.g., HIV infection, long-term immunosuppressive therapy, primary immunodeficiency diseases); or (5) incomplete clinical or laboratory data.

Clinical data were collected by trained healthcare professionals from the hospitals' medical information systems.

Sample size was determined via the Obuchowski method based on an expected AUC of 0.80 versus a null of 0.70, with *α* = 0.05 (two-sided), a statistical power of 1- *β* = 0.80, and a 1:1 case-to-control ratio. The calculated minimum was 62 subjects per group. Accounting for a 10% rate of non-compliant specimens or missing data, the final sample size for each group was set at at least 68 subjects.

### Definitions and classification

In accordance with established guidelines for PTB and CAP, all enrolled children were classified into predefined diagnostic categories based on a combination of microbiological, laboratory, radiographic, and clinical findings. The specific diagnostic criteria were as follows:

Confirmed PTB was defined as follows (13, 14): (1) presence of clinical signs, symptoms, or chest radiological findings suggestive of PTB; and (2) identification of MTB by conventional culture or molecular test (e.g., GeneXpert MTB/RIF) from at least one representative clinical specimen [sputum, gastric lavage, or bronchoalveolar lavage fluid (BALF)].

Unconfirmed PTB (13, 14) was defined as the presence of clinical or chest radiological signs suggestive of PTB (lymphadenopathy, cavitation, miliary pattern, airways narrowing because of compression, etc.), combined with at least one of the following criteria: (1) immunological evidence of MTB infection, including a positive tuberculin skin test (TST) with an induration diameter ≥ 10 mm or a positive IGRA; and/or (2) documented exposure to MTB confirmed by contact tracing.

The diagnosis of CAP was based on the “Guideline for the Diagnosis and Treatment of Community-Acquired Pneumonia in Children (2019)” (12), in accordance with the definitions used in previous studies (15–17). To assign etiological subgroups, respiratory specimens (nasopharyngeal swab, sputum or BALF) were subjected to multiplex fluorescent PCR and standard bacterial culture. We defined pathogens by all of the following criteria: (1) For bacteria, only sputum or BALF cultures yielding predominant growth (> 10^5^ CFU/mL for sputum, > 10^4^ CFU/mL for BALF); positive bacterial PCR from nasopharyngeal swabs alone were not used to assign bacterial CAP. (2) For viruses, a positive nasopharyngeal PCR was accepted as causative only when the detected virus was consistent with the seasonal epidemiology and the patient's clinical-radiological findings, while excluding other more likely bacterial etiologies. (3) For *Mycoplasma pneumoniae*, diagnosis required positive PCR and/or serum-specific IgM antibodies > 1:160. Based on these criteria, all CAP cases were stratified into four subgroups: viral CAP (Viral-CAP), bacterial CAP (Bacterial-CAP), *Mycoplasma pneumoniae* CAP (MP-CAP), and mixed CAP (Mixed-CAP) confirmed co-infection with viruses, bacteria, and/or *Mycoplasma pneumoniae*.

### Sample collection and measurement

Venous blood samples were obtained from each participant within 24 h after hospital admission. Samples were centrifuged at 3,000 rpm for 10 min and stored at −80 °C until analysis. Serum S100A8/A9 concentrations were determined using a Human Calprotectin enzyme-linked immunosorbent assay (ELISA) kit (Abcam, UK). ELISAs were performed in serial batches at regular intervals (every 4–6 weeks) throughout the study period. All batches met the acceptance criteria with an inter-batch coefficient of variation (CV) < 10%, confirming stable assay performance. Each sample was measured in duplicate, and the mean values were used for analysis. Researchers performing all laboratory tests were blinded to the final diagnosis.

### Statistical analysis

Statistical analyses were conducted using IBM SPSS (version 27.0) and R software (version 4.4.3). No missing data were present and no imputation methods were applied. Continuous variables were presented as median with interquartile range (IQR) and analyzed using Mann–Whitney U tests or Kruskal–Wallis tests. The chi-squared test and Fisher's exact test were used for categorical variables, with results presented as frequency (percentage). Receiver operating characteristic (ROC) curve analysis, both unadjusted and age-adjusted (using multivariable logistic regression), was used to evaluate predictive performance. The optimal cut-off value was determined by the Youden index (sensitivity + specificity - 1). Delong's test compared ROC curves, with Bonferroni correction for multiple comparisons. The lower and upper cutoffs from subgroup-specific ROC analyses in the discovery cohort were used to define stratified risk zones and were evaluated as fixed thresholds in the independent validation cohort. Calibration curves were employed to estimate model calibration, and decision curve analysis (DCA) was performed to evaluate clinical utility (R packages: pROC version 1.18.5 for ROC and Delong's test; rms version 6.8–2 for calibration curves; rmda version 1.6 for DCA). *p* < 0.05 was considered statistically significant.

## Results

### Baseline characteristics

A total of 1,629 children were enrolled in this study, including a discovery cohort of 1,092 participants and a validation cohort of 537 participants. The discovery cohort comprised 217 patients with PTB, 775 with CAP, and 100 HC. The validation cohort consisted of 96 patients with PTB, 390 with CAP, and 51 HC (Figure 1). Notably, significant age differences existed between PTB and CAP groups (Table 1). However, within each disease group (i.e., PTB discovery vs. PTB validation, and CAP discovery vs. CAP validation), the discovery and validation cohorts were comparable in age, which ensured the consistency of baseline characteristics and laid a solid foundation for the cross-validation of subsequent diagnostic efficacy results.

**Figure 1:**
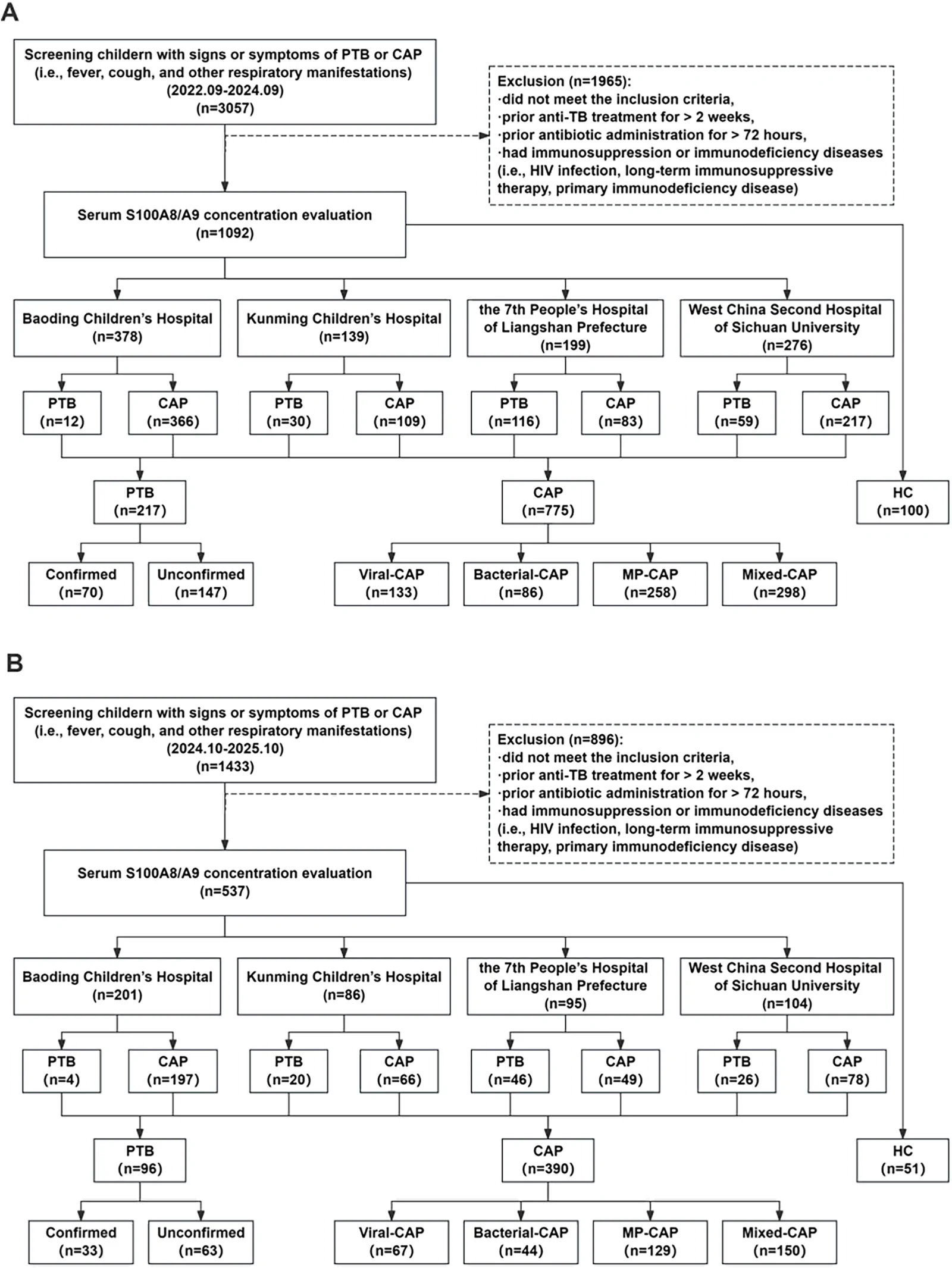
Flowchart of the screening process for children with symptoms of pulmonary tuberculosis (PTB) or community-acquired pneumonia (CAP) in two cohorts. (**A**) Discovery cohort; (**B**) Validation cohort. Both panels outline inclusion and exclusion criteria, participant numbers, hospital sites, diagnostic categories, and CAP subtypes. Each branch shows specific sample sizes, illustrating the selection process and classification into confirmed or unconfirmed PTB, viral, bacterial, *Mycoplasma pneumoniae* (MP), mixed CAP, or healthy controls (HC).

**Table 1:** Patient characteristics and serum S100A8/A9 levels.

	Discovery cohort (*n* = 1,092)				Validation cohort (*n* = 537)			
Variables	PTB	CAP	HC	*p* value	PTB	CAP	HC	*p* value
Number	217	775	100		96	390	51	
Age (years)	9 (5, 12)	5 (3, 7)	5 (3, 9)	<0.001	8 (5, 12)	5 (2, 8)	4 (2, 9)	<0.001
< 5 (n,%)	54 (24.9)	374 (48.3)	41 (41.0)		22 (22.9)	189 (48.5)	29 (56.9)	
5–18 (n,%)	163 (75.1)	401 (51.7)	59 (59.0)		74 (77.1)	201 (51.5)	22 (43.1)	
Gender (Male/Female)	118/99	424/351	51/49	0.922	58/38	229/161	34/17	0.547
TST (n,%)				<0.001				<0.001
Positive	164 (75.6)	30 (3.9)	——		70 (72.9)	14 (3.6)	——	
Negative	53 (24.4)	745 (96.1)	——		26 (27.1)	376 (96.4)	——	
IGRA (n,%)				<0.001				<0.001
Positive	168 (77.4)	8 (1.0)	——		74 (77.1)	3 (0.8)	——	
Negative	49 (22.6)	767 (99.0)	——		22 (22.9)	387 (99.2)	——	
MTB culture or Xpert MTB/RIF (n,%)								
Positive	70 (32.3)	——	——		33 (34.4)	——	——	
Negative	147 (67.7)	——	——		63 (65.6)	——	——	
S100A8/A9 (ng/mL)	599 (395, 1,276)	4,681 (2,401, 8,071)	241 (151, 408)	<0.001	615 (375, 1,101)	4,613 (2,456, 8,091)	251 (151, 521)	<0.001

TST positive, induration diameter ≥ 10 mm.

### Analysis of S100A8/A9 levels between PTB and HC

In both the discovery and validation cohorts, serum S100A8/A9 concentrations in the PTB group were significantly higher than HC group (both *p* < 0.001) (Figures 2A,D). ROC curve analysis in the discovery cohort yielded an AUC of 0.857 (95% CI: 0.815–0.900) for differentiating PTB from HC, with a sensitivity of 90.8% and a specificity of 66.0% at a cutoff of 307 ng/mL. After adjustment for age, the AUC improved to 0.885 (95% CI: 0.848–0.922), with a sensitivity of 75.1% and specificity of 86.0% (Figure 2B; Supplementary Table S1). These findings were confirmed in the validation cohort, where the AUC values before and after age adjustment were 0.814 (95% CI: 0.744–0.883) and 0.856 (95% CI: 0.795–0.917), respectively (Figure 2E, Supplementary Table S1). Calibration plots and DCA were presented in Supplementary Figure S1.

**Figure 2:**
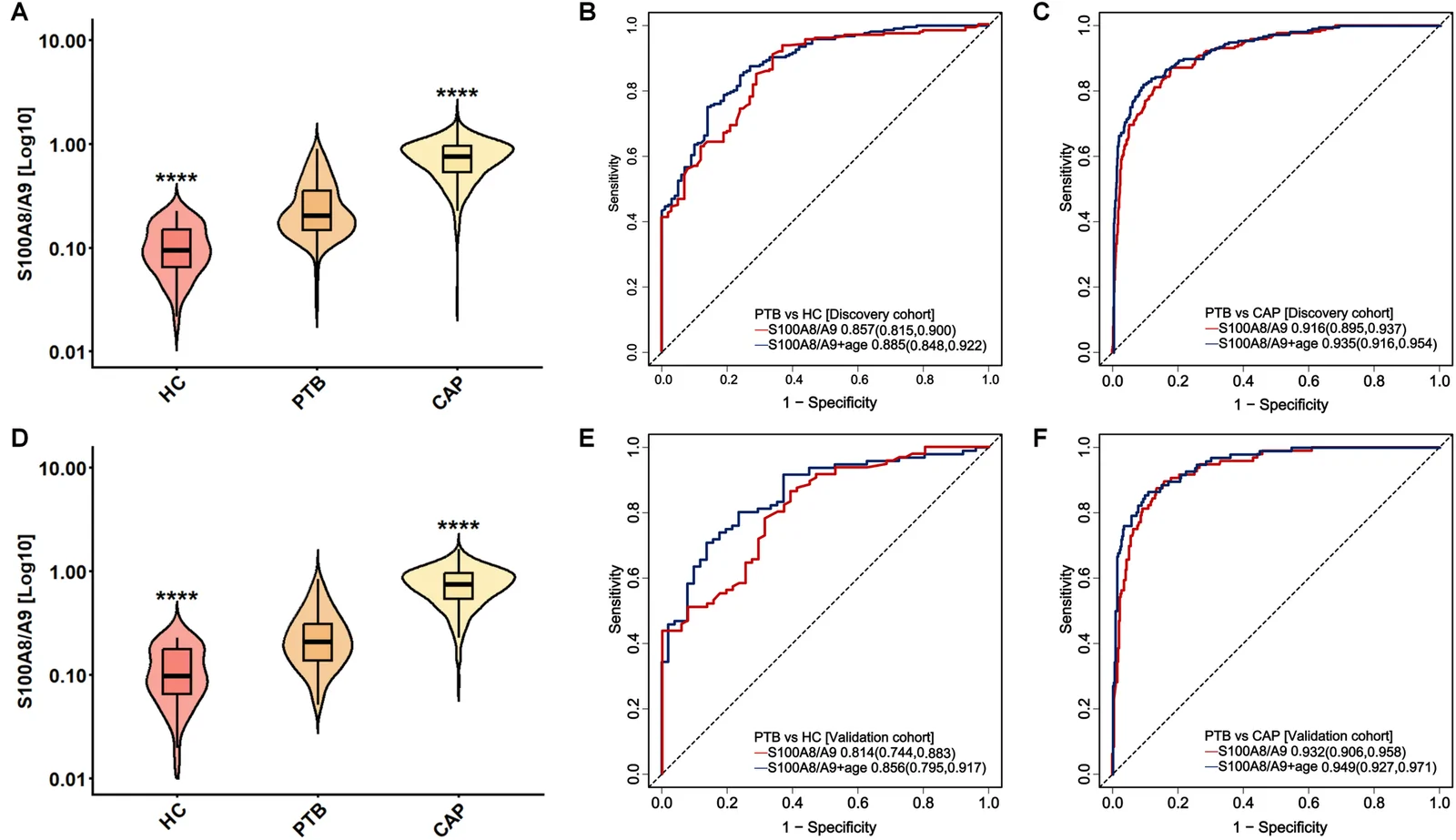
Diagnostic value of serum S100A8/A9 for discriminating PTB from CAP and HC in the discovery and validation cohorts. (**A**) Serum S100A8/A9 concentrations in HC, PTB and CAP groups in the discovery cohort. (**B,C**) ROC curves of serum S100A8/A9 for discriminating PTB from HC (**B**) and CAP (**C**) in the discovery cohort. (**D**) Serum S100A8/A9 concentrations in HC, PTB, CAP groups in the validation cohort. (**E,F**) ROC curves of serum S100A8/A9 for discriminating PTB from HC (**E**) and CAP (**F**) in the validation cohort. Data are shown as median with interquartile range. ****p < 0.0001.

### Diagnostic value of S100A8/A9 for differentiating PTB from CAP

In the discovery cohort, serum S100A8/A9 concentrations were significantly lower in children with PTB than in those with CAP (median 599 ng/mL vs. 4,681 ng/mL, *p* < 0.001) (Table 1; Figure 2A). ROC analysis demonstrated that S100A8/A9 had satisfactory discriminatory efficacy for distinguishing PTB from CAP, with an AUC of 0.916 (95% CI: 0.895–0.937). At the optimal cut-off value of 1,876 ng/mL, the sensitivity and specificity reached 87.1% and 81.8%, respectively. Adding age to the model modestly improved the AUC to 0.935 (95% CI: 0.916–0.954) (Figure 2C; Supplementary Table S2).

In the validation cohort, S100A8/A9 yielded an AUC of 0.932 (95% CI: 0.906–0.958), with a sensitivity of 87.5% and specificity of 86.4% at the cut-off value of 1,687 ng/mL (Figure 2F; Supplementary Table S2), which was comparable to that in the discovery cohort. After age adjustment, the AUC further increased to 0.949 (95% CI: 0.927–0.971) (Figure 2F; Supplementary Table S2).

To validate the generalizability of the discovery-derived threshold, we applied the optimal cut-off value of 1,876 ng/mL to the independent validation cohort. Under this cut-off value, the overall accuracy for differentiating PTB from CAP was 84.0%, with a sensitivity of 89.6% and specificity of 82.6% (Table 2).

**Table 2:** Validation of serum S100A8/A9-based predictive model for differentiating PTB from CAP.

Group	Actual	Predictive PTB	Predictive HC/CAP	Overall Accuracy	Sensitivity	Specificity
S100A8/A9 307 ng/mL						
PTB	96	80	16	75.5%	83.3%	60.8%
HC	51	20	31			
S100A8/A9 1,876 ng/mL						
PTB	96	86	10	84.0%	89.6%	82.6%
CAP	390	68	322			
S100A8/A9 970 ng/mL						
PTB	96	69	27	79.1%	71.9%	89.6%
Viral	67	7	60			
S100A8/A9 1,926 ng/mL						
PTB	96	87	9	87.1%	90.6%	79.5%
Bacterial	44	9	35			
S100A8/A9 1,876 ng/mL						
PTB	96	86	10	92.0%	89.6%	93.8%
MP	129	8	121			
S100A8/A9 1,517 ng/mL						
PTB	96	81	15	86.2%	84.4%	87.3%
Mixed	150	19	131			

PTB, pulmonary tuberculosis; CAP, community-acquired pneumonia; MP, *Mycoplasma pneumonia.*

Calibration curves showed satisfactory agreement between predicted and observed outcomes in both cohorts, and DCA curves yielded positive net clinical benefit across clinically relevant threshold probabilities, suggesting that the model guided by S100A8/A9 would effectively differentiate PTB from CAP (Supplementary Figure S2).

To further evaluate the diagnostic performance of S100A8/A9, we compared S100A8/A9 alone and in combination with neutrophil-lymphocyte ratio (NLR) and C-reactive protein (CRP). Owing to incomplete data for these conventional markers, this analysis was performed on merged discovery and validation cohorts (PTB, *n* = 93; CAP, *n* = 1,165). Multivariable logistic regression adjusting for age, NLR, and CRP showed that S100A8/A9 remained independently associated with PTB (OR = 0.443, 95% CI: 0.362–0.544, *p* < 0.001), whereas neither NLR (*p* = 0.268) nor CRP (*p* = 0.394) retained significance (Supplementary Table S3). ROC analysis revealed that S100A8/A9 achieved an AUC of 0.897, significantly higher than NLR (0.524) and CRP (0.612) (both *p* < 0.001) (Supplementary Table S4). Regarding combined models, adding NLR to S100A8/A9 modestly increased the AUC to 0.909, whereas further addition of age and/or CRP yielded no significant improvement, with the full four-marker model reaching an AUC of 0.924 (Supplementary Table S4).

### Subgroup analysis: PTB vs. pathogen-specific CAP subgroups

We also performed subgroup analysis comparing PTB with four pathogen-specific CAP subgroups (Viral-CAP, Bacterial-CAP, MP-CAP, and Mixed-CAP). In both the discovery and validation cohorts, children with PTB had the lowest serum S100A8/A9 levels among all subgroups (all *p* < 0.001), with the most significant difference observed between PTB and MP-CAP (Fig. S3A, D). ROC curve analysis showed that the AUC values for differentiating PTB from each CAP subgroup ranged from 0.863 (PTB vs. Viral-CAP) to 0.954 (PTB vs. MP-CAP) (Supplementary Figure S3B,C,E,F, Supplementary Table S5), indicating that S100A8/A9 has good discriminatory efficacy for PTB regardless of CAP etiology, especially for the clinically confusing PTB vs. MP-CAP distinction. Subgroup-specific calibration plots and DCA demonstrated the model reliability and positive net clinical benefit (Supplementary Table S4–S7).

Additionally, S100A8/A9 levels were comparable between confirmed PTB and unconfirmed PTB (median 631 ng/mL vs. 589 ng/mL, *p* = 0.329) (Figure 3A). For confirmed PTB versus CAP, S100A8/A9 achieved an AUC of 0.923 (95% CI: 0.898–0.948). Similar discriminative performance was observed in unconfirmed PTB versus CAP (AUC = 0.920, 95% CI: 0.900–0.939) (Figures 3B,C). Calibration plots and DCA were shown in Supplementary Figure S8.

**Figure 3:**
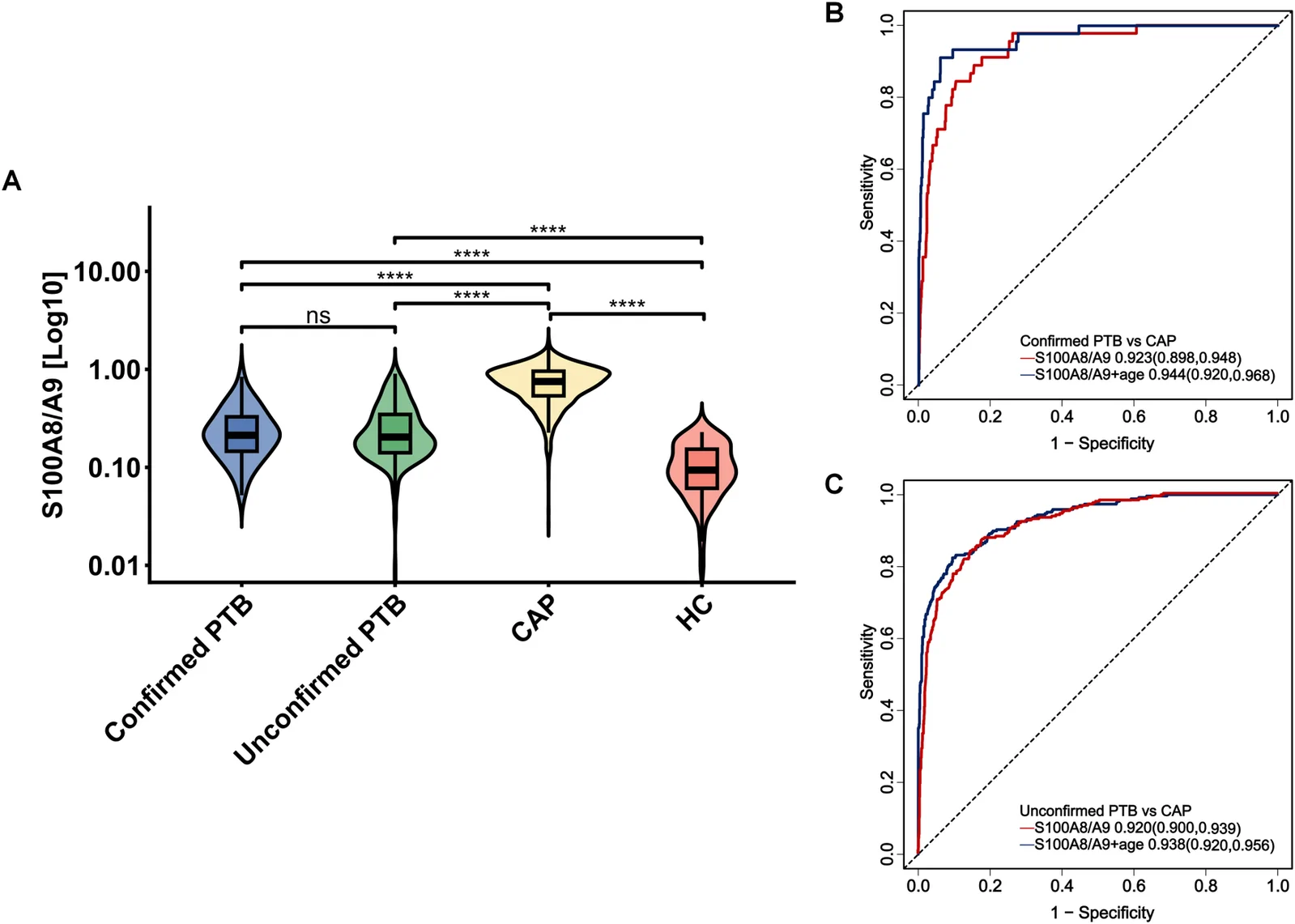
Diagnostic value of serum S100A8/A9 for discriminating confirmed and unconfirmed PTB from CAP. **(A)** Serum S100A8/A9 concentrations in confirmed PTB, unconfirmed PTB, CAP and HC groups. **(B,C)** ROC curves of serum S100A8/A9 for discriminating confirmed PTB **(B)** and unconfirmed PTB **(C)** from CAP. Data are shown as median with interquartile range. ****p < 0.0001.

### S100A8/A9 concentrations in age and diagnosis groups

Across both age groups (under 5 years and 5–18 years), PTB had significantly lower serum S100A8/A9 levels than all CAP subcategories, yet markedly higher than HC (*p* < 0.001) (Supplementary Table S6). Interestingly, S100A8/A9 concentrations increased with age in all CAP subgroups,whereas the PTB group showed the opposite trend, with younger patients having higher levels than older ones (836 ng/mL vs. 579 ng/mL, *p* = 0.006) (Supplementary Table S6). However, the capability of S100A8/A9 to differentiate PTB from CAP was superior in the older age group (AUC 0.955 vs. 0.838, *p* < 0.001) (Supplementary Table S7,S8).

### Age-stratified diagnostic value of S100A8/A9 based on ROC-derived cutoffs

Age-stratified boundaries for differentiating PTB from CAP were determined based on the extreme pathogen-specific optimal cutoffs identified in the discovery cohort (Supplementary Table S7,S8). For clinical convenience, the thresholds were rounded to 1,000 and 2,500 ng/mL in children under 5 years, and the thresholds were rounded to 1,000 and 1,900 ng/mL in children aged 5–18 years. These boundaries were then applied to the entire validation cohort. Figure 4 presented the distribution of PTB and CAP cases in each interval. In children under 5 years, although CAP cases dominated across all strata (CAP, *n* = 189; PTB, *n* = 22), the PTB distribution showed a decreasing trend from <1,000 ng/mL stratum to the > 2,500 ng/mL stratum. The descriptive misclassification rate for this age group was 13.9%. In children aged 5–18 years, levels below 1,000 ng/mL were predominantly observed in PTB cases, with a positive predictive value (PPV) for PTB of 92.1%. Within the 1,000-1,900 ng/mL interval, a shift toward CAP was observed, with CAP comprising 61.5% of cases (PPV for CAP 61.5%). When concentrations exceeded 1,900 ng/mL, CAP was overwhelmingly dominant, representing 96.8% of cases (PPV for CAP 96.8%), with only 3.2% of cases being PTB [negative predictive value (NPV) for PTB 96.8%]. The descriptive misclassification rate for this age group was 4.4%. To illustrate the impact of disease prevalence on predictive values, we calculated PPVs and NPVs across a range of PTB prevalence estimates (5%–40%) using the likelihood ratios derived from the validation cohort (Supplementary Table S9).

**Figure 4:**
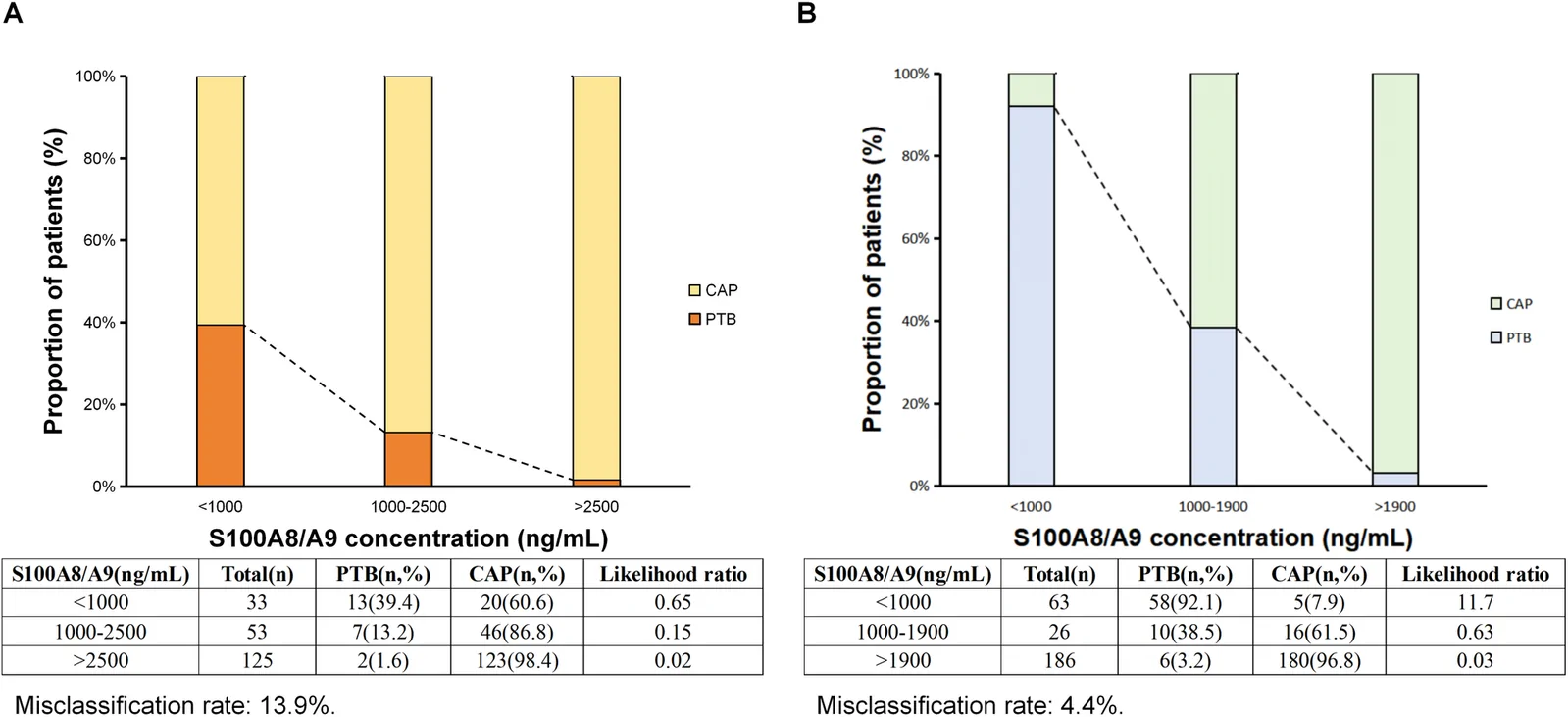
Age-stratified diagnostic value of serum S100A8/A9 for discriminating PTB from CAP using ROC-derived cutoffs. **(A)** Children aged < 5 years; **(B)** Children aged 5–18 years.

## Discussion

In this multicenter prospective study, we identified serum S100A8/A9 as a promising biomarker for distinguishing pediatric PTB from CAP, with diagnostic performance consistently validated across two independent cohorts. These findings not only underscore the potential clinical utility of S100A8/A9 particularly in resource-limited regions, but also provide insights into the inflammatory signatures underlying different disease states.

S100A8/A9 is a heterodimeric calcium-binding protein that serves as a key mediator of innate immunity and inflammatory responses (8). Active PTB is characterized by chronic granulomatous inflammation, which drives the activation and infiltration of inflammatory cells, leading to increased release of S100A8/A9 into the circulation. In contrast, HC have no obvious inflammatory response, resulting in low baseline serum S100A8/A9 levels. Accordingly, our results showed that S100A8/A9 levels were significantly higher in PTB patients than in HC.

A key finding of this study is the favorable discriminatory efficacy of serum S100A8/A9 in distinguishing PTB from CAP. As expected, serum S100A8/A9 concentrations in the PTB group were significantly lower than those in the CAP group (*p* < 0.001), yielding AUCs of 0.916 and 0.932 in the discovery and validation cohorts, respectively. Although the WHO has set an optimal sensitivity threshold of 66% for pediatric tuberculosis tests (18), currently available WHO-endorsed tests fall short of this target, with reported sensitivities of only 42%–63% in children (19, 20). This diagnostic gap highlights the potential value of S100A8/A9, which in our study maintained a sensitivity of > 80% and specificity of nearly 90% across two independent cohorts in distinguishing PTB from CAP.

This diagnostic performance may be attributable to differences in the nature and severity of inflammatory responses between PTB and CAP. S100A8/A9 is abundant in neutrophils and monocytes, accounting for about 45% of cytoplasmic proteins in neutrophils (8). As a damage-associated molecular pattern (DAMP), it amplifies inflammatory responses by recruiting and activating additional neutrophils, creating a positive feedback loop. Bacterial and *Mycoplasma pneumoniae* infections are characterized by extensive neutrophilic infiltration (21–23) Upon activation, neutrophils release massive amounts of S100A8/A9, and the feedback loop may further elevate circulating S100A8/A9 levels. In Viral-CAP, although neutrophilic infiltration is not the dominant feature, the acute inflammatory response also triggers robust S100A8/A9 release, with the magnitude of increase correlating with the severity of acute infection (24, 25). In contrast, PTB is a chronic granulomatous disease featuring predominant monocyte and lymphocyte infiltration and relatively limited neutrophil involvement (26, 27). This diminished neutrophil activation and chronic inflammatory activation may be consistent with the lower S100A8/A9 levels in children with PTB.

Notably, the elevated S100A8/A9 levels reported in advanced adult TB (28) are not contradictory to our findings. Those results represent the chronic, damage-associated phase of TB, where sustained inflammation drives S100A8/A9 expression upward. However, our study exclusively enrolled children in the early active infection phase (≤ 2 weeks of treatment), a stage when the chronic, neutrophil-driven pathology characteristic of advanced TB has not yet peaked. Thus, the apparent discrepancy between our pediatric early-stage cohort and the adult late-stage model likely reflects the temporal dynamics of S100A8/A9 expression.

When compared with conventional inflammatory biomarkers (NLR and CRP), S100A8/A9 showed superior performance in differentiating PTB from CAP, and multivariable adjustment confirmed its independent association with PTB. This advantage is probably attributed to the non-specific nature of NLR and CRP, which are elevated in most acute inflammatory conditions but cannot reliably distinguish between TB-related chronic inflammation and CAP-induced acute responses (29, 30). Adding NLR to S100A8/A9 modestly improved the AUC, but the clinical significance of this modest gain remains uncertain. By contrast, adding CRP to S100A8/A9 yielded no further benefit, which may be related to the overlapping roles of CRP and S100A8/A9 in acute inflammatory responses. Importantly, the subcohort analyzed here comprises only those PTB cases with available NLR and CRP data, and its limited sample size may constrain the generalizability of the findings. Further validation in larger prospective cohorts is warranted.

Subgroup analysis further demonstrated that S100A8/A9 maintained good discriminatory efficacy between PTB and all pathogen-specific CAP subgroups, especially for the clinically confusing distinction between PTB and MP-CAP (AUC = 0.954). Despite the application of strict interpretative criteria, residual misclassification cannot be entirely excluded. In this study, the Mixed-CAP subgroup was most susceptible to colonization-related misclassification, which may potentially underestimate the true differences in S100A8/A9 between Mixed-CAP and PTB. Nevertheless, this limitation does not compromise the primary finding that S100A8/A9 exhibits favourable performance in distinguishing PTB from overall CAP.

A notable finding is that S100A8/A9 showed lower performance in children under 5 years (AUC = 0.838) than in older children (AUC = 0.955). The particularly low sensitivity (57.4%) against Viral-CAP in this age group warrants careful consideration. We propose two explanations for this age-dependent attenuation. First, the immune systems in children under 5 years are incompletely developed (31), and MTB infection tends to elicit an acute-like inflammatory response rather than typical chronic granulomas seen in older children, leading to higher baseline S100A8/A9 release. Second, Viral-CAP itself induces epithelial-derived S100A8/A9 expression, further confounding the distinction from PTB. Critically, despite the modest performance in children under 5 years, S100A8/A9 still demonstrated significant differences between PTB and CAP (all *p* < 0.01), and the median levels in PTB remained lower than those in Viral-CAP (583 ng/mL vs. 1,690 ng/mL). These findings support its use as an adjunct to conventional diagnostic methods.

Moreover, S100A8/A9 levels exhibited a concentration-dependent trend in discriminating PTB from CAP. In children aged 5–18 years, concentrations below 1,000 ng/mL were predominantly observed in PTB cases, whereas levels exceeding 1,900ng/mL were overwhelmingly associated with CAP. In the intermediate range (1,000-1,900 ng/mL), CAP became the predominant diagnosis. These results indicate that in older children, S100A8/A9 may serve as an adjunctive biomarker for differential diagnosis between PTB and CAP, particularly for those with suspected PTB but negative or delayed GeneXpert/microbiological results. In children under 5 years, although CAP cases outnumbered PTB cases across all S100A8/A9 strata, the proportion of PTB cases showed a decreasing trend as S100A8/A9 levels increased. However, given the limited number of PTB cases in this age group, these findings require validation in larger pediatric cohorts. Collectively, S100A8/A9 is not a substitute for microbiological confirmation, but rather an early immunological complement that may help stratify the pre-test probability of PTB versus CAP during the critical window before definitive results are available. Importantly, in patients with high clinical suspicion of TB or severe illness, S100A8/A9 results should not be used in isolation to exclude TB, as elevated calprotectin levels may occur in severe TB or TB–bacterial coinfection. S100A8/A9 results should always be interpreted in conjunction with clinical features, chest imaging, and molecular assays. Of note, the PPVs and NPVs reported here reflect the prevalence of PTB in our study population (approximately 20%), which is determined by the assembled case mix. In clinical settings with different PTB prevalence, the predictive values would vary accordingly. Further studies are needed to validate its diagnostic value across different age groups and outpatient settings.

This study has several strengths. First, this multicenter prospective study, encompassing four hospitals across three Chinese provinces, provided a large sample size with independent discovery and validation cohorts, ensuring external validity. Second, methodological rigor was ensured by consecutive and standardized enrollment, trained data collection, and blinded laboratory assays. Third, pathogen-stratified CAP subgroups and bacteriology-confirmed PTB revealed S100A8/A9's diagnostic value in diverse clinical scenarios.

Several limitations should be acknowledged. First, the application of strict diagnostic criteria to our cohort restricted enrollment to cases with definitive diagnoses, potentially overestimating diagnostic performance and limiting generalizability to clinical practice. Second, despite S100A8/A9's favorable performance in differentiating PTB from CAP, the diagnostic uncertainty in the lower concentration range (< 1,000 ng/mL) suggests that combining it with other markers (e.g., IFN-*γ*, IP-10) may further improve accuracy. Additionally, the development of a reliable nomogram incorporating clinical, inflammatory, and radiological features is warranted to enable individualized risk prediction. Finally, while our multi-center design enhances representativeness, the reported PPV and NPV are prevalence-dependent and should be interpreted with caution when applied to populations with different disease burdens.

In conclusion, this study demonstrates that serum S100A8/A9 is a promising non-invasive biomarker for distinguishing pediatric PTB from CAP, exhibiting a concentration-dependent diagnostic pattern.

## Data Availability

The datasets presented in this article are not publicly available due to ethical restrictions related to protecting participant anonymity and confidentiality. Requests to access the datasets should be directed to Adong Shen, 13370115087@163.com.
