# Supplementary material for: S100A8/A9 as a biomarker for differentiating pulmonary tuberculosis from community-acquired pneumonia in children: a multicenter cohort study in China
Source: Front Pediatr. 2026 Oct 1;14:1910674. doi: 10.3389/fped.2026.1910674 (PMC13670915; doi:10.3389/fped.2026.1910674)
Supplement: Supplementary file 1 [file Supplementaryfile1.docx]

**Table S1.** Diagnostic efficacy of S100A8/A9 levels for distinguishing PTB from HC in discovery and validation cohort.

| **Discovery cohort** | | | |  | **Validation Cohort** | | | |  |
| --- | --- | --- | --- | --- | --- | --- | --- | --- | --- |
| **AUC (95%CI)** | **Cut-off value** | **Sensitivity** | **Specificity** | ***p* value** | **AUC (95%CI)** | **Cut-off value** | **Sensitivity** | **Specificity** | ***p* value** |
| **S100A8/A9 (ng/mL)** |  |  |  |  | **S100A8/A9 (ng/mL)** |  |  |  |  |
| 0.857 (0.815, 0.900) | 307 | 0.908 | 0.660 | Ref. | 0.814 (0.744, 0.883) | 293 | 0.865 | 0.608 | Ref. |
| **S100A8/A9 + Age** | | | | | **S100A8/A9 + Age** | | | | |
| 0.885 (0.848, 0.922) | — | 0.751 | 0.860 | <0.001 | 0.856 (0.795, 0.917) | — | 0.863 | 0.708 | <0.001 |

**Table S2.** Diagnostic efficacy of S100A8/A9 levels for distinguishing PTB from CAP in discovery and validation cohort.

| **Discovery cohort** | | | |  | **Validation Cohort** | | | |  |
| --- | --- | --- | --- | --- | --- | --- | --- | --- | --- |
| **AUC (95%CI)** | **Cut-off value** | **Sensitivity** | **Specificity** | ***p* value** | **AUC (95%CI)** | **Cut-off value** | **Sensitivity** | **Specificity** | ***p* value** |
| **S100A8/A9 (ng/mL)** |  |  |  |  | **S100A8/A9 (ng/mL)** |  |  |  |  |
| 0.916 (0.895, 0.937) | 1876 | 0.871 | 0.818 | Ref. | 0.932 (0.906, 0.958) | 1687 | 0.875 | 0.864 | Ref. |
| **Age (years)** |  |  |  |  | **Age (years)** |  |  |  |  |
| 0.727 (0.686, 0.768) | 7.05 | 0.576 | 0.785 | <0.001 | 0.694 (0.629, 0.758) | 9.05 | 0.438 | 0.877 | <0.001 |
| **S100A8/A9 + Age** | | | | | **S100A8/A9 + Age** | | | | |
| 0.935 (0.916, 0.954) | — | 0.823 | 0.910 | 0.001 | 0.949 (0.927, 0.971) | — | 0.865 | 0.892 | 0.022 |

**Table S3. Multivariable logistic regression of inflammation biomarkers for distinguishing PTB from CAP.**

| **Variable** | **Odds Ratio** | **95% CI** | ***p* value** |
| --- | --- | --- | --- |
| Age (years) | 1.450 | 1.345, 1.563 | <0.001 |
| S100A8/A9 (ng/mL) | 0.443 | 0.362, 0.544 | <0.001 |
| NLR | 1.064 | 0.953, 1.189 | 0.268 |
| CRP (mg/mL) | 1.006 | 0.992, 1.020 | 0.394 |

PTB = 1 vs. CAP = 0; NLR, neutrophil-lymphocyte ratio; CRP, C-reactive protein.

**Table S4. Diagnostic efficacy of biomarker combinations for distinguishing PTB from CAP.**

| **Variable** | **PTB (n = 93)** | **CAP (n = 1165)** | ***p* value** | **AUC (95%CI)** | **Cut-off**  **value** | **Sensitivity** | **Specificity** | ***p* value** |
| --- | --- | --- | --- | --- | --- | --- | --- | --- |
| S100A8/A9 (ng/mL) | 583 (351, 1131) | 4641 (2436, 8071) | <0.001 | 0.897 (0.856, 0.939) | 1616 | 0.828 | 0.859 | ref |
| Age (years) | 12 (7, 14) | 5 (2, 7) | <0.001 | 0.816 (0.756, 0.876) | 9.1 | 0.710 | 0.881 | 0.034 |
| NLR | 1.56 (0.91, 2.84) | 1.84 (0.95, 3.08) | 0.448 | 0.524 (0.466, 0.581) | 1.783 | 0.602 | 0.522 | <0.001 |
| CRP (mg/mL) | 2.35 (0.10, 17.08) | 7.65 (1.75, 20.2) | <0.001 | 0.612 (0.543, 0.681) | 1.005 | 0.495 | 0.803 | <0.001 |
| S100A8/A9 + Age | - | - | - | 0.922 (0.887, 0.956) | - | 0.774 | 0.949 | 0.087 |
| S100A8/A9 + NLR | - | - | - | 0.909 (0.868, 0.949) | - | 0.839 | 0.914 | 0.020 |
| S100A8/A9 + CRP | - | - | - | 0.898 (0.857, 0.939) | - | 0.796 | 0.912 | 0.635 |
| S100A8/A9 + Age + NLR | - | - | - | 0.922 (0.888, 0.957) | - | 0.774 | 0.954 | 0.080 |
| S100A8/A9 + Age + CRP | - | - | - | 0.924 (0.890, 0.958) | - | 0.763 | 0.958 | 0.052 |
| S100A8/A9 + NLR + CRP | - | - | - | 0.909 (0.869, 0.950) | - | 0.839 | 0.922 | 0.017 |
| S100A8/A9 + Age + NLR + CRP | - | - | - | 0.924 (0.890, 0.959) | - | 0.774 | 0.954 | 0.056 |

NLR, neutrophil-lymphocyte ratio; CRP, C-reactive protein.

**Table S5.** Diagnostic efficacy of S100A8/A9 levels for distinguishing PTB from CAP subgroups in discovery and validation cohort.

|  | **S100A8/A9** | | | |  | **Age** | | | | |  | **S100A8/A9 + Age** | | | |
| --- | --- | --- | --- | --- | --- | --- | --- | --- | --- | --- | --- | --- | --- | --- | --- |
| **PTB vs** | **AUC**  **(95%CI)** | **Cut-off value**  **(ng/mL)** | **Sensitivity** | **Specificity** |  | **AUC**  **(95%CI)** | **Cut-off value**  **(years)** | **Sensitivity** | **Specificity** | ***p* value** |  | **AUC**  **(95%CI)** | **Sensitivity** | **Specificity** | ***p* value** |
| **Discovery cohort** | |  |  |  |  |  |  |  |  |  |  |  |  |  |  |
| Viral-CAP | 0.863  (0.824, 0.902) | 970 | 0.696 | 0.902 |  | 0.843  (0.802, 0.885) | 5.35 | 0.714 | 0.835 | 0.522 |  | 0.931  (0.906, 0.956) | 0.783 | 0.955 | <0.001 |
| Bacterial-CAP | 0.895  (0.857, 0.933) | 1926 | 0.871 | 0.767 |  | 0.771  (0.715, 0.827) | 5.35 | 0.714 | 0.756 | <0.001 |  | 0.918  (0.886, 0.949) | 0.839 | 0.837 | 0.119 |
| MP-CAP | 0.954  (0.937, 0.972) | 1876 | 0.871 | 0.942 |  | 0.603  (0.549, 0.657) | 10.25 | 0.415 | 0.884 | <0.001 |  | 0.955  (0.937, 0.972) | 0.848 | 0.961 | 0.815 |
| Mixed-CAP | 0.913  (0.888, 0.937) | 1517 | 0.811 | 0.866 |  | 0.770  (0.728, 0.812) | 7.85 | 0.567 | 0.866 | <0.001 |  | 0.935  (0.915, 0.955) | 0.807 | 0.909 | 0.007 |
| **Validation cohort** | |  |  |  |  |  |  |  |  |  |  |  |  |  |  |
| Viral-CAP | 0.883  (0.830, 0.936) | 1032 | 0.750 | 0.881 |  | 0.822  (0.758. 0.887) | 4.59 | 0.781 | 0.806 | 0.155 |  | 0.930  (0.891, 0.968) | 0.854 | 0.896 | 0.049 |
| Bacterial-CAP | 0.925  (0.881, 0.970) | 1691 | 0.875 | 0.841 |  | 0.797  (0.724, 0,871) | 6.25 | 0.646 | 0.932 | 0.005 |  | 0.962  (0.936, 0.989) | 0.875 | 0.932 | 0.037 |
| MP-CAP | 0.967  (0.946, 0.989) | 1809 | 0.896 | 0.954 |  | 0.549  (0.468, 0,631) | 11.40 | 0.333 | 0.915 | <0.001 |  | 0.967  (0.946, 0.988) | 0.896 | 0.946 | 0.982 |
| Mixed-CAP | 0.925  (0.893, 0.958) | 1687 | 0.875 | 0.860 |  | 0.730  (0.662, 0.798) | 6.25 | 0.646 | 0.740 | <0.001 |  | 0.949  (0.924, 0.973) | 0.865 | 0.893 | 0.050 |

**Table S6.** Serum S100A8/A9 levels of children with different pathogens in different ages.

|  | **0-5 years** | | **5-18 years** | | ***p* value** |
| --- | --- | --- | --- | --- | --- |
| **Group** | **Number** | **S100A8/A9 (ng/mL)** | **Number** | **S100A8/A9 (ng/mL)** |  |
| HC | 70 | 236 (151, 402) | 81 | 241 (156, 436) | 0.881 |
| PTB | 76 | 836 (417, 1801) | 237 | 579 (371, 1011) | 0.006 |
| CAP | 563 | 3679 (1739, 7211) | 602 | 5545 (3229, 8816) | <0.001 |
| Viral | 157 | 2663 (1335, 5673) | 43 | 5103 (2551, 9319) | 0.001 |
| Bacterial | 89 | 4011 (1642, 7471) | 41 | 4375 (2396, 8943) | 0.203 |
| MP | 67 | 5181 (3239, 8559) | 320 | 5595 (3491, 8586) | 0.400 |
| Mixed | 250 | 4140 (1837, 7535) | 198 | 5623 (2871, 9299) | <0.001 |
| *p* value |  | <0.001 |  | <0.001 |  |

**Table S7.** Diagnostic efficacy of S100A8/A9 levels for distinguishing PTB and CAP in children aged < 5 years.

| **PTB vs** | **AUC (95%CI)** | **Cut-off value**  **(ng/mL)** | **Sensitivity** | **Specificity** |
| --- | --- | --- | --- | --- |
| **HC** | 0.927 (0.879, 0.975) | 412 | 0.870 | 0.829 |
| **CAP** | 0.838 (0.787, 0.889) | 2508 | 0.907 | 0.634 |
| **Viral-CAP** | 0.777 (0.702, 0.852) | 970 | 0.574 | 0.874 |
| **Bacterial-CAP** | 0.840 (0.768, 0.911) | 2553 | 0.907 | 0.632 |
| **MP-CAP** | 0.928 (0.874, 0.981) | 2593 | 0.907 | 0.867 |
| **Mixed-CAP** | 0.850 (0.797, 0.904) | 2508 | 0.907 | 0.663 |

**Table S8.** Diagnostic efficacy of S100A8/A9 levels for distinguishing PTB and CAP in children aged 5-18 years.

| **PTB vs** | **AUC (95%CI)** | **Cut-off value**  **(ng/mL)** | **Sensitivity** | **Specificity** |
| --- | --- | --- | --- | --- |
| **HC** | 0.829 (0.769, 0.888) | 284 | 0.933 | 0.627 |
| **CAP** | 0.955 (0.936, 0.974) | 1867 | 0.896 | 0.923 |
| **Viral-CAP** | 0.953 (0.923, 0.983) | 1967 | 0.896 | 0.900 |
| **Bacterial-CAP** | 0.927 (0.878, 0.976) | 1922 | 0.896 | 0.897 |
| **MP-CAP** | 0.961 (0.942, 0.980) | 1872 | 0.896 | 0.953 |
| **Mixed-CAP** | 0.951 (0.930, 0.973) | 1791 | 0.883 | 0.915 |

**Table S9.** Positive and negative predictive values of S100A8/A9 at varying PTB prevalence estimates (children aged 5-18 years).

| **PTB Prevalence** | **PPV for PTB**  **(if S100A8/A9 < 1000 ng/mL)^a^** | **NPV for PTB**  **(if S100A8/A9 > 1900 ng/mL)^b^** |
| --- | --- | --- |
| 5% | 38% | > 99% |
| 10% | 57% | 99% |
| 20% (Study population) | 74% | 99% |
| 30% | 83% | 98% |
| 40% | 89% | 98% |

a: Calculated using the positive likelihood ratio (LR+) of S100A8/A9 < 1000 ng/mL for PTB (LR+ = 11.7, derived from the validation cohort). b: Calculated using the negative likelihood ratio (LR-) of S100A8/A9 > 1900 ng/mL for excluding PTB (LR- = 0.03).

**Fig S1. Calibration and decision curves of the S100A8/A9-alone and age-adjusted models for PTB vs. HC in the discovery (A, B) and validation (C, D) cohorts.**

**Fig S2. Calibration and decision curves of the S100A8/A9-alone and age-adjusted models for PTB vs. CAP in the discovery (A, B) and validation (C, D) cohorts.**

**Fig S3. Diagnostic value of serum S100A8/A9 for discriminating PTB from CAP subgroups (based on pathogen) in discovery and validation cohorts.**

(A) Serum S100A8/A9 concentrations in PTB and CAP subgroups in the discovery cohort. (B-C) ROC curves of serum S100A8/A9 (B) and serum S100A8/A9 combined with age (C) for discriminating PTB from CAP subgroups in the discovery cohort. (D) Serum S100A8/A9 concentrations in PTB and CAP subgroups in the validation cohort. (E-F) ROC curves of serum S100A8/A9 (E) and serum S100A8/A9 combined with age (F) for discriminating PTB from CAP subgroups in the validation cohort. Data are shown as median with interquartile range. *****p* < 0.0001.

**Fig S4. Calibration and decision curves of the S100A8/A9-alone and age-adjusted models for PTB vs. Viral-CAP in the discovery (A, B) and validation (C, D) cohorts.**

**Fig S5. Calibration and decision curves of the S100A8/A9-alone and age-adjusted models for PTB vs. Bacterial-CAP in the discovery (A, B) and validation (C, D) cohorts.**

**Fig S6. Calibration and decision curves of the S100A8/A9-alone and age-adjusted models for PTB vs. MP-CAP in the discovery (A, B) and validation (C, D) cohorts.**

**Fig S7. Calibration and decision curves of the S100A8/A9-alone and age-adjusted models for PTB vs. Mixed-CAP in the discovery (A, B) and validation (C, D) cohorts.**

**Fig S8. Comparison of calibration and decision curves between the S100A8/A9-alone and S100A8/A9 + age models in the confirmed and unconfirmed PTB vs. CAP cohorts. (A-B) confirmed PTB vs. CAP; (2) unconfirmed PTB vs. CAP.**
